# Cognitive and hippocampal changes weeks and years after memory training

**DOI:** 10.1038/s41598-022-11636-4

**Published:** 2022-05-12

**Authors:** Anne Cecilie Sjøli Bråthen, Øystein Sørensen, Ann-Marie G. de Lange, Athanasia M. Mowinckel, Anders M. Fjell, Kristine B. Walhovd

**Affiliations:** 1 Department of Psychology, Center for Lifespan Changes in Brain and Cognition, University of Oslo, 0317 Oslo, Norway; 2 Department of Radiology and Nuclear Medicine, Oslo University Hospital, Oslo, Norway; 3 Present Address: LREN, Department of Clinical Neurosciences, Centre for Research in Neurosciences, Lausanne University Hospital (CHUV), University of Lausanne, Lausanne, Switzerland

**Keywords:** Long-term memory, Hippocampus, Cognitive ageing, Cognitive neuroscience, Neuroscience, Psychology, Ageing, Brain imaging

## Abstract

While immediate effects of memory-training are widely reported in young and older adults, less is known regarding training-dependent hippocampal plasticity across multiple intervention phases, and long-term maintenance of such. Here, 157 healthy young and older adults underwent a training-intervention including two 10 weeks periods of episodic-memory training, separated by two 2 weeks periods of no training. Both age groups showed improvements on a criterion task, which prevailed after 3 years. When compared to the reference condition of no training, relative increases in hippocampal volume were observed after the training across age groups, which were maintained after 10 weeks periods of no training. However, there was age-group dependent temporal variation with respect to timing of effects. Hippocampal volume of the training group did not differ from that of a passive control-group 3 years after the intervention. The young showed an immediate near-transfer effect on a word-association task. We show that training-gains on memory performance can prevail for at least 3 years. Memory training can induce increases in hippocampal volume immediately after the intervention and after months. Episodic-memory training can produce transfer effects to a non-trained memory task in young adults. However, maintained effects on hippocampal volume beyond 10 weeks are uncertain, and likely require continuous training.

## Introduction

Episodic-memory function is crucial for storage and recollection of personal experiences^1^, but weakens with increasing age^2–4^. The hippocampus is pivotal for episodic-memory function^5–8^, and also shows accelerating volumetric decline from around the age of 60 years onwards^9,10^. Increasing attention is given to memory-training as a possible approach to attenuate such neurocognitive deterioration. Benefits to cognitive abilities from memory training are well-documented in both young and older adults^11–13^, and maintenance of these have been observed even years after training interventions^14,15^. Neural changes with training interventions have also been observed, but these have been reported to be dependent on continued training, and possibly more so than cognitive improvements, with retraction of the neural gains within weeks or months of training cessation^16–20^. Age-related changes in hippocampal volume have been related to episodic-memory decline in healthy adults^21–24^, suggesting a possible relationship between the two during episodic-memory training. However, as long-term maintenance of neural training benefits seems possibly limited relative to retainment of cognitive improvements, it is unknown to what extent any hippocampal plasticity associated with memory training can persist over months and years, and whether maintenance of training-induced brain-changes is required to uphold cognitive training-effects for longer time periods.

While transfer effects to non-targeted cognitive processes are undoubtedly desirable, results regarding training-induced transfer are inconclusive^25,26^, and their possible dependence on training-induced changes to brain substrates common to criterion and transfer tasks have been discussed^27,28^. Although transfer effects from training interventions are mixed, participants in a training study that aimed to improve episodic memory performance with the usage of mnemonics were observed to additionally improve their performance on a word pair task^29^. This indicates that episodic-memory training can improve memory functions beyond the trained task.

A theoretical framework elaborated by Lövdén et al.^51^ propose that cognitive improvements can indeed occur without brain changes, by distinguishing between the terms flexibility and plasticity. Flexibility refers to the capacity of optimalization within the current constraints of the brain without depending on structural changes, which would be a true plastic response. According to the framework, if solving a cognitive task is within the already achieved neural resources of a person, no brain change would be needed to appropriately solve the cognitive demand at hand^30^. In this manner, while the hippocampus is crucial for episodic memory function, hippocampal structural changes may not be required for immediate and long-term cognitive training effects. Both cognitive behavioral and structural hippocampal training changes over time are addressed in the present study, where we assessed a large sample undergoing periods of memory training and rest, at multiple time-points with MRIs and cognitive tests, for a period of up to 3 years.

The healthy sample consisted of 57 adults in their 20 s and 100 adults in their 70 s. The intervention comprised two 10 weeks training periods (A) aiming to improve episodic memory by implementing the mnemonic technique Method of Loci (MoL)^31^, separated by two 10 weeks passive periods (B) in an ABAB/BABA-design. Training gains were tested with an episodic-memory task measuring written recall of 100 words. Participants underwent magnetic resonance imaging (MRI) scans of the brain and neuropsychological tests before and after each training/no-training period. Additionally, MRI and neuropsychological tests were conducted at a 3 years follow-up assessment.

We asked four main questions:

(1) Does the training increase memory performance, and can effects be observed 3 years after the intervention? We hypothesized that there would be a specific effect of the training on the criterion task and that this would persist up to 3 years after the intervention. (2) Does the training lead to an increase or maintenance of hippocampal volume relative to that observed with no training, and can such effects be observed 3 years after the intervention? We hypothesized that the training would increase or maintain hippocampal volume relative to that observed with no training, and that this would be training-dependent, so that effects would no longer be observed years after the intervention. (3) Is there a relationship between memory and hippocampal volume changes following training? We hypothesized that a positive relationship would be found. (4) Is there a transfer effect on a non-trained memory-task, and if so, does it relate to the 100-words test performance, and does it persist after cessation of the intervention? Based on mixed results on transfer after training in previous training studies, we did not have firm grounds to expect transfer effects, but if found, we hypothesized that these too would persist after cessation of the intervention.

## Methods

### Sample

**Figure 1:**
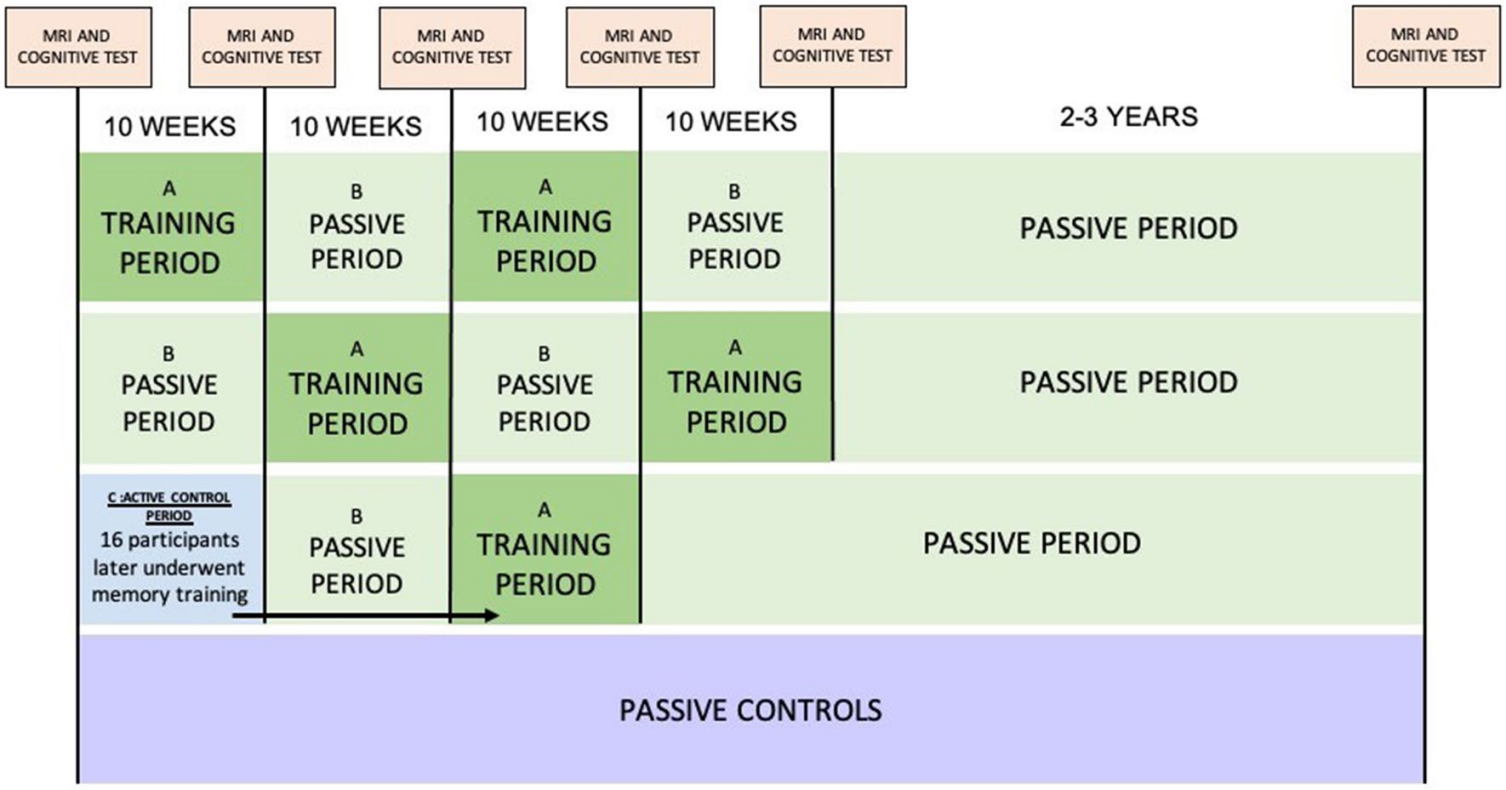
Design. Memory-training groups: the memory trainers were allocated to either an ABAB (top row) condition or a BABA (second row) condition. The training intervention was equal for both conditions. Active control group (third row); after participating in the active control group, participants were offered to undergo a 10 weeks passive period followed by a training period, resulting in a CBA design. 16 participants (4 young, 12 older) proceeded to train. Passive control group (bottom row); a long-term passive control group was tested and scanned at two time points with no intervention, with a similar interval between baseline and follow-up as the rest of participants.

The sample was from the project “Neurocognitive Plasticity” at the Center for Lifespan Changes in Brain and Cognition (LCBC), Department of Psychology, University of Oslo. All methods were carried out in accordance with relevant guidelines and regulations. The procedures were approved by the Regional Ethical Committee of Southern Norway, and written, informed consent was obtained from all participants. Participants were recruited through newspaper and webpage adverts, and were screened with a health interview. Participants were required to be in or around their 20 s and 70 s, healthy adults, right-handed, fluent Norwegian speakers, and have normal or corrected to normal vision and hearing. Exclusion criteria were history of injury or disease known to affect central nervous system (CNS) function, including neurological or psychiatric illness or serious head trauma, being under psychiatric treatment, use of psychoactive drugs known to affect CNS functioning, and MRI contraindications. All scans were evaluated by a neuroradiologist and deemed free of significant injuries or conditions. For inclusion in the study, participants were required to score ≥ 26 on the Mini Mental State Examination (MMSE)^32^ and have scores less than 2 standard deviations below mean on the 5 min delayed recall subtest of the California Verbal Learning Test II (CVLT)^33^. Three individuals were excluded based on these criteria. All participants further had to achieve an IQ above 85 on the Wechsler Abbreviated Scale of Intelligence (WASI)^34^. A total of 143 participants (53 young and 88 older) fulfilled the inclusion criteria. We have previously reported that memory improvements were specific to the memory training group, i.e., no training effects were observed in an active control group^35^. The active control group took part in weekly meetings centered on popular science topics, and completing home exercises on these, of similar magnitude as the memory-training group. Based on the zero effect in the control group, all participants from the active control group were offered to undergo 10 weeks of memory training after completing the control condition. A total of 4 young and 12 older participants from the active control group opted in and completed the training intervention after 10 weeks as active controls. They are included in all the subsequent analyses of the “memory training group”. Hence, a total of 57 young and 100 older participants are included in the training group. For an overview of the design, see Fig. 1. For demographics of the groups, see Tables 1 and 2.

**Table 1:** Demographics of young and older training groups at all-time points, separated by group allocation according to the ABAB/BABA design.

		Tp0 Pre rest 1 (only for BABA)		Tp1 Pre intervention 1		Tp2 Post intervention 1		Tp3 Pre intervention 2		Tp4 Post intervention 2		TP5 Post rest 2 (only for ABAB)		TP6 follow-up	
		Mean	SD	Mean	SD	Mean	SD	Mean	SD	Mean	SD			Mean	SD
BABA	Sex	15F/18 M		14F/16 M		10F/9 M		8F/7 M		7F/5 M				9F/7 M	
	Age	26.3	3.08	26.5	3.04	26.9	3.16	27.6	2.89	27.4	2.99			21.1	3.10
Start rest young	Interval (years)	0	0	0.207	0.017	0.422	0.007	0.627	0.025	0.843	0.009			2.61	0.826
	MMSE	29.3	1.01	29.3	1.05	29.3	1.08	29.7	0.617	29.7	0.651			29.1	1.09
	IQ	114	9.42	114	8.25	117	7.03	118	5.77	119	5.73			118	7.36
	Hippocampus V	8498	483	8427	538	8406	533	8456	509	8475	535			8308	516
	ICV	1,568,544	142,940	1,560,001	138,770	1,535,560	122,619	1,528,742	115,206	1,545,548	107,988			1,552,498	118,837

	Sex	37F/17 M		35F/16 M		31F/11 M		31F/11 M		31F/10 M				27F/11 M	
	Age	73.3	3.15	73.6	3.20	73.9	3.31	74.0	3.21	74.2	3.21			76.3	3.11
Start rest older	Interval (years)	0	0	0.208	0.007	0.419	0.008	0.631	0.008	0.845	0.008			2.98	0.51
	MMSE	28.9	1.11	28.9	1.10	29.0	1.0	28.9	1.03	28.9	1.03			28.8	1.08
	IQ	118	10.4	118	10.5	119	8.87	119	8.87	119	8.98			120	10.5
	Hippocampus vol	7381	664	7347	709	7358	704	7422	673	7331	675			7154	707
	ICV	1,513,891	152,239	1,510,386	151,621	1,488,502	136,470	1,490,586	139,568	1,488,149	136,479			1,519,384	155,138

ABAB	nex			27F/20 M		19F/15 M		18F/14 M		18F/10 M		16F/10 M		15F/12 M	
	Age			26.1	3.20	26.6	3.21	26.8	3.18	27.3	3.13	27.4	3.13	29.4	3.52
Start train young	Interval (years)			0	0	0.212	0.026	0.420	0.006	0.640	0.040	0.837	0.012	3.00	0.832
	MMSE			29.2	0.770	29.2	0.834	29.2	0.847	29.2	0.749	29.2	0.749	29.0	1.11
	IQ			110	9.23	112	9.11	112	9.23	112	9.56	112	8.70	116	8.77
	Hippocampus vol			8522	793	8454	796	8414	763	8383	714	8418	742	8405	843
	ICV			1,566,036	158,453	1,549,683	171,993	1,543,301	167,490	1,541,285	157,707	1,556,008	171,793	1,548,876	154,726

	Sex			28F/31 M		25F/21 M		21F/21 M		21F/20 M		20F/20 M		24F/8 M	
	Age			73.0	2.62	73.4	2.62	73.8	2.57	74.0	2.59	74.1	2.56	75.8	2.86
Start train older	Interval (years)			0	0	0.209	0.008	0.420	0.011	0.631	0.018	0.838	0.007	2.92	0.803
	MMSE			28.6	1.38	28.8	1.31	28.8	1.36	28.8	1.35	28.8	1.35	28.8	1.13
	IQ			121	10.9	123	10.1	123	10.2	123	10.3	123	10.4	122	11.6
	Hippocampus vol			7377	867	7369	834	7350	827	7359	847	7351	854	7120	727
	ICV			1,564,626	169,810	1,553,857	169,738	1,566,882	158,851	1,560,586	155,607	1,562,928	156,926	1,588,817	142,828

Values on hippocampal volume refers to both hemispheres.

**Table 2:** Demographics of the young and older active control groups. Time points 1 and 2 correspond to the assessments conducted before and after the active control period. Time points 3 and 4 correspond to the assessments conducted before and after the optional training period offered to the participants after completion of the active control condition.

Active ctrl		Tp1 Pre active control		Tp2 Post active control		Tp3 Pre train 1		Tp4 Post train 1		TP6 follow-up	
		Mean	SD	Mean	SD	Mean	SD	Mean	SD	Mean	SD
	Sex	17F/2 M		11F/2 M		3F/2 M		2F/2 M		3F/2 M	
Active control young	Age	26.5	3.13	26.8	3.18	25.4	2.69	26.2	2.76	29.7	3.37
	Interval (years)	0	0	0.211	0.004	0.419	0.008	0.632	0.006	3.03	0.063
	MMSE	29.1	1.15	29.5	0.660	29.6	0.548	29.8	0.5	28.8	1.30
	IQ	111	5.72	112	5.27	109	4.53	111	0.816	120	7.91
	Hippocampus V	8498	762	8640	844	8394	702	8451	902	8376	739
	ICV	1,499,071	117,576	1,521,741	128,359	1,530,523	123,021	1,532,709	141,940	1,510,608	132,486

	Sex	13F/7 M		12F/7 M		8F/4 M		8F/4 M		9F/4 M	
Active control older	Age	73.7	2.81	73.7	2.78	73.8	3.11	74.0	3.11	76.4	2.67
	Interval (years)	0	0	0.212	0.003	0.424	0.004	0.634	0.006	2.96	0.117
	MMSE	28.2	1.50	28.3	1.45	27.9	1.68	27.9	1.68	28.8	0.689
	IQ	122	6.48	121	7.69	121	6.50	121	6.50	123	8.24
	Hippocampus V	7391	523	7385	567	7481	638	7537	700	6996	479
	ICV	1,559,762	151,059	1,562,482	154,694	1,578,514	136,230	1,597,752	126,875	1,578,779	182,051

Values on hippocampal volume refer to both hemispheres.

All participants were invited to undergo a follow-up MRI scan and neuropsychological test after an extended time of no intervention. A total of 48 young and 83 older participants accepted to return for the long-term follow-up assessment. The interval from baseline to follow-up did not significantly differ between the age-groups (mean interval young participants: 2.90 years (SD: 0.80, range 1.8–4.6 years), mean interval older participants: 2.97 years (SD: 0.61, range 1.8–4.4 years)). See Supplementary Fig. S2 for distribution of the follow-up interval and Supplementary Fig. S3 for time since training at all observations.

At follow-up, 83 participants (47 young, 36 older) were additionally pooled from an ongoing longitudinal observation study sample at LCBC^36^ and included in the design as long-term passive controls. Control participants were required to have MRI scans and cognitive test data from two time points with a similar time interval as the participants from the training group. The passive controls were selected merely based on age (mean age young: 24.3, SD: 3.07, mean age older: 71.9, SD: 3.36) and availability of scans (mean interval young: 3.80 years, SD: 0.51, mean interval older: 2.33 years, SD: 0.47). All participants who fulfilled these criteria, in addition to the inclusion criteria of the present study, were included as passive controls regardless of any other results obtained from previous assessments.

Independent sample t-tests showed no significant differences at baseline between the young and older group in terms of sex (t(211.54) = − 0.14, *p* = 0.86, male/female young: 41/59, male/female old: 55/78) or years of education (t(225.01) = 1.52, *p* = 0.12, mean young: 15.53 years, SD: 2.78, mean older; 15.01, SD: 3.22). As expected, younger adults had higher MMSE score (t(229.48) = 3.99, *p* < 0.001, mean score young: 29.2, SD: 0.93, mean score older: 28.62, SD: 1.3) and CVLT scores (t(224.59) = 10.12, *p* < 0.001, mean score young: 14.04 words, SD: 2.22, mean score older: 10.22 words, SD: 3.51). The older participants had higher IQ than the younger (t(225.04) = − 6.35, *p* < 0.001, mean score young: 111.6, SD: 8.8, mean score older: 119.6, SD: 10.2), perhaps suggesting some selection effect. 19 young and 14 older participants dropped out after the first training period. To test for attrition effects commonly observed in longitudinal studies^37^, a drop-out analysis was carried out by correlating number of assessment (MRI and neuropsychological tests) time points completed with several cognitive measures collected at baseline; (IQ, CVLT 30 min free recall, MMSE, and years of education). Given expected age-differences in all variables except from IQ, age was used as a covariate for the analyses of MMSE, CVLT, and years of education. The results showed that number of follow-up assessments correlated positively with score on MMSE (r = 0.19, *p* = 0.004), IQ (r = 0.32, *p* < 0.001), and CVLT 30 min free recall (r = 0.21, *p* = 0.001), while no relationship was observed with years of education. An independent sample t-test was conducted to investigate any differences between those who underwent more than one assessment (regardless of how many) and the drop-outs. Here, IQ for those who remained in the study was higher (t(43.37) = 4.44, *p* < 0.001), while the groups did not differ in terms of MMSE, education, CVLT 30 min free recall, or education.

### Experimental design

Pools of around 20 participants in their 20 s and their 70 s were recruited at a time, and the participants were assigned to undergo either a memory-training intervention or an active control intervention (see descriptions below). The participants in the memory-training group were allocated in an ABAB/BABA cross-over design with two 10 weeks training periods interspersed with two 10 weeks periods of rest. All periods were preceded and followed by a neurocognitive test assessment and an MRI scan (a total of 5 assessments). The first memory-training group (ABAB allocation = 34 young, 46 older) started with 10 weeks of memory training and moved on to a subsequent 10 weeks rest period with no intervention. This rest period was followed by a second 10 weeks training period, and then a final 10 weeks rest period. In an inverse manner, the second memory-training group (BABA allocation = 19 young adults, 42 older adults) started with 10 weeks of rest and moved on to the first training period followed by a second rest period and a subsequent training. Given that both training groups (ABAB and BABA) underwent the same training intervention, merely in an opposite order, all participants who underwent memory training are referred to as participants of the memory-training group in the analyses below. An active control group (13 young adults, 19 older adults) completed scanning prior to, and immediately following the 10 weeks control intervention. As participants for the study were recruited through ads advertising for a memory training project, we considered that the most ethical approach was to offer memory training to all participants, including those who were assigned to the active control group. From the active control group, 16 participants (4 young, 12 older) underwent 10 weeks of memory training 10 weeks after the active control condition. For design illustration, see Fig. 1 above (Methods section).

Finally, regardless of the total number of 10 weeks periods completed in the memory-training intervention or as active controls, all participants were invited to undergo a follow-up MRI-scan and neuropsychological test after an extended time of no intervention. A total of 48 young and 83 elderly opted in. As the main focus of the study was to investigate the long-term maintenance of benefits from memory training, the sixth time point was chosen to be carried out after 3 years, as this was the longest interval possible within the project plan.

### Memory training intervention and active control condition

The memory training involved learning and practicing the mnemonic technique MoL, specifically aiming to improve episodic memory performance. The training intervention included two periods of weekly in-class course sessions for 10 weeks, in addition to 8 weekly home assignments to be carried out online throughout the training periods. Independent sample t-tests showed a significant age group difference in terms of number of tasks completed between the young and older group (t(109.21) = − 0.847, *p* =  < 0.001, mean tasks completed young: 36.48, mean tasks completed old: 77.43). We aimed to create groups of around 12 participants, but due to challenges regarding the fixed schedules for the meetings for some participants, the group size ranged from 4 to 16 participants (mean group size = 9.3, SD = 2.9). Each course session had a duration of approximately 1 h. The assignments involved tasks of word lists to be memorized by applying the MoL. The first group session included a presentation of the project and an introduction to the MoL with instructions, and an initial word-list task consisting of 15 words. The participants were instructed to create a mental “travel route” through a familiar building such as their home. Each participant defined a personalized route of 15 places (“loci”) through their own house, such as the mailbox, main door, hallway, kitchen or similar, in what they personally considered to be in a logical order. To apply the technique, the participants were instructed to visualize the first word of the word list at the first place on the travel route, the second word on the list at the second place, and so forth. If the words were sufficiently visualized with their corresponding places along the travel route, a “mental walk” along the route facilitated the recollection of the word lists. The research fellow leading the group session was available for questions and provided repetition or any needed clarifications to ensure that all participants were able to apply the technique. The following weekly group sessions included updating of the strategy, clarification of instructions, and a new word-list task, which was increased by five words each week to ensure a continuous challenge. However, the lists were increased by ten words during the last 3 weeks of the memory training. The participants were instructed not to practice any memory training in the 10 weeks passive periods. All participants received the same assignments. However, the participants were encouraged to individually adjust the difficulty level of the tasks both in class and of the home assignments, with the aim of achieving a challenging but manageable training level across all the participants. Individual adjustments included increasing or decreasing the number of words, performing the tasks within individual time limits, and recollection of the word lists in reverse order.

The active control intervention involved popular scientific lectures, followed by group discussions, once a week during 10 weeks. Similarly to the training group, eight home assignments were sent out weekly to be completed online. However, the home assignments of the active control group involved tasks related to the weekly lecture topics instead of the targeted MoL tasks. Contact with staff, group meetings and the number of tasks were matched between the training group and the active control group.

The data collection was on-going and continuous for all conditions simultaneously. This ensured that participants from all experimental groups were scanned and tested interchangeably and thus reducing the possibility of group differences with regards to the assessment and scanning conditions. The lectures in the active control program were carried out by several lecturers. To be able to organize this, the active control intervention was only carried out once. The sample size of the active control group is therefore based on the number of participants who signed up during the relevant dates preceding the active control intervention. Although group assignments based on date do not comply with suggested criteria for randomization of participants^38^, practical considerations forced a compromise due to the extensive data collection with strict time intervals and assessments locked to specific dates across 40 weeks.

### Data acquisition and processing

A Siemens Skyra 3 T MRI scanner with a 24-channel head-coil was used (Siemens Medical Solutions; Erlangen, Germany). The volumetric analyses were based on a 3D T1-weighted Magnetization Prepared Rapid Gradient Echo (MP-RAGE) pulse sequence (TR/TE/TI = 2300/2.98/850 ms, FA = 8°, matrix size = 192 × 192, 176 sagittal slices, voxel size = 1.0 × 1.0 × 1.0 mm^3^, field of view = 240 mm) with a total duration of 9 min and 50 s. The data was processed with the FreeSurfer software recon-all pipeline (version 6.0; http://surfer.nmr.mgh.harvard.edu/). To extract reliable volumetric estimates for each time point, images were automatically processed with the longitudinal stream^39^. Specifically, an unbiased within-subject template space and image^40^ is created using robust, inverse consistent registration^41^. Several processing steps, such as skull stripping, Talairach transforms, atlas registration as well as spherical surface maps and parcellation are then initialized with common information from the within-subject template, significantly increasing reliability and statistical power^39^. *All scans were* manually quality checked for motion artifacts and segmentation output. Three older participants were excluded due to low quality of the MRI-scans.

## Statistical analyses and outcome measures

All statistical analyses were performed in R^42^ with mixed models fitted using the “nlme” package^43^.

### Does the intervention increase memory performance and can effects be observed three years after the cessation of intervention?

Memory performance was measured by the number of correct written recalls of a word list consisting of 100 nouns (100-words test) administrated on the neuropsychological test sessions at baseline, and after each memory training period, rest period, and at follow-up. The words of the word-lists differed at all six time-points. Each version contained nouns matched for word frequency in Norwegian newspapers and magazines, using the Frequency lists for Norwegian spoken and written language, provided by The Text laboratory at the University of Oslo^44^. The participants were given five minutes to memorize the word list, followed by ten minutes to recall as many words as possible. The extensive length of the lists was chosen to avoid ceiling effects. We have previously shown significant change in this measure, hereafter termed “memory performance” as a result of the memory training intervention in a largely overlapping sample, which was not due to test–retest effects, as it was seen primarily with memory training, rather than across test–retest after passive or active control periods^35^. To confirm this in the present sample, we estimated the effect of the training intervention on subsequent memory performance using nonlinear mixed models^45^.

#### Estimation of training and retest effects

All participants with two or more time points who had undergone at least one training period were included, and the number of words from the 100-words test recalled at each time point was treated as a continuous outcome variable. The main goal of the modelling was to estimate how training affects memory both after a training period, and after several weeks and years. We defined $\beta_{ }$ as an immediate effect after a training period, and included $x_{1}$ and $x_{2}$ as dummy variables indicating whether the participant had completed exactly one or exactly two training periods prior to the given time point, respectively. To estimate how the training effects prevailed as time since training increased, we included an exponential function in the model, on the form $\left( {x_{1} \beta_{1} + x_{2} \beta_{2} } \right)e^{{ - \lambda {\Delta }t}}$, where ${\Delta }t$ is the time since last training at a given time-point, and $\lambda$ is an exponential decay parameter describing the extent to which the training effect is retained with time. Since ${\Delta }t = 0$ at time-points immediately following a training period, and $e^{{ - \lambda {\Delta }t}} = 1$ when ${\Delta }t = 0$, $\beta_{1}$ and $\beta_{2}$ estimated the immediate effect of the first and the second training on memory performance. The training effect was allowed to interact with age by estimating separate training effects and decay parameters for each age group, see Table 3.

**Table 3:** Parameters of interest in nonlinear mixed model estimating the effect of training on memory.

	Young	Old
Effects of first training period	$\beta_{1,young}$	$\beta_{1,older}$
Effects of second training period	$\beta_{2, young}$	$\beta_{2,older}$
Exponential decay over time	$\lambda_{young}$	$\lambda_{older}$

Main effects for sex and age group were also included in the model. To ensure that separation of the age groups for further analyses of the training benefits on memory performance was statistically justified, we tested for an interaction between age group and time. This was done by fitting a simpler model than the one explained above, where the age group x time interaction was not included in the model. We also tested for an interaction between age group and training status, this time keeping the age group x time interaction in the model but removing age group x training status interaction. Both interactions were significant (*p* < 0.001). Potential memory decline with increasing age during the course of the study was incorporated with a variable encoding the difference between the participant’s age at a given time point, and the overall mean age in the age group, with a slope estimated for each age group. Retest effects were accounted for by including a dummy variable indicating whether the participant had taken the test one or more times prior to the given time point. Further information regarding estimation of retest effects, aging effects and other covariates are illustrated in the provided R code (see SI). Finally, a random intercept term for each participant was included, to account for correlation between repeated measurements of the same individuals. For interpreting the long-term effect of training on memory, the value of the exponential term was computed over a dense grid of time intervals ${\Delta }t$ between 0 and 5 years, at the estimated values of $\beta_{1}$ and $\lambda$ for each age group separately. Confidence intervals for these predictions were computed using 10,000 samples from a nonparametric case bootstrap coupled with individual residual bootstrap^46^. See Supplementary Figs. S1, S2 and S3, for distribution of time intervals training and follow-up. See Supplementary Fig. S4 for estimation of long-term effects on memory performance up to 10 years after training. The bootstrap function can be found in the provided code repository (see SI).

### Does the training intervention lead to an increase or maintenance of hippocampal volume relative to that observed with no training, and are such effects observed years after intervention?

The datasets used were slightly different from the ones used in the previous sections, as different participants had missing values for hippocampal volume and memory performance. All participants with hippocampal volumes were included (57 young, 97 older).

#### Short-term effect of training on hippocampal atrophy

To estimate the short-term memory training effects (immediately after training and after 10 passive weeks) on hippocampal volume, we defined a categorical variable encoding the training status of the participants at each time point of MRI scan and neuropsychological assessment. The status “post train” was used when the participant had trained immediately prior to the scan (that is, after an “A” period in the ABAB/BABA design), “post rest” when the participant had completed a training period 10 weeks prior to the scan (that is, after a “B” period in the ABAB/BABA design), and “baseline” otherwise (where the participant neither had trained before the current nor the previous measurement). We fitted a linear mixed model with hippocampal volume as outcome variable, and the interaction between training status and age group as the main effect of interest. Effects of age group, time since baseline in each age group, total intracranial volume (ICV), and sex were also included as covariates. Random intercept and slopes of time for each participant were also included. “Baseline” was used as reference level, and hence the effects of training status “post train” and training status “post rest” were estimated. The former can be interpreted as the immediate effect of a training period on hippocampal volume, while the latter can be interpreted as the remaining effect of training on hippocampal volume 10 weeks after training was completed. As conducted for the cognitive analyses (“Does the training intervention lead to an increase or maintenance of hippocampal volume relative to that observed with no training, and are such effects observed years after intervention?”section) we tested for an interaction between age group and time by not including the age group x time interaction in the model. We also tested for an interaction between age group and training status, this time keeping the age group x time interaction in the model but removing the age group x training status interaction. Both interactions were significant (*p* < 0.001 and *p* = 0.012, respectively).

#### Long-term effect of training on hippocampal atrophy

To estimate the long-term effect of training on hippocampal volume 3 years after the training completion, we first compared the hippocampal volume trajectories throughout the whole study period (from initiation of training to follow-up time point) of participants who trained once or more (training group) to participants who never trained (long-term passive control group). A linear mixed model with hippocampal volume as outcome variable was fitted, the effect of interest being the different effect of aging on hippocampal volume between the training and control group, separately for each age group. We used all time points prior to the first training session, as well as the follow-up. With a linear mixed model, the slope estimates are unbiased, independent of the number of time points used per participant. Hence, including all available time points per participant, even though this leads to heterogeneous data, increases statistical power without impacting the validity of the study. To this end, a three-way interaction between time since baseline, age group, and training/control status was included. All lower-order terms related to this interaction were included. In addition, ICV and sex were included as covariates, and random intercepts and slopes of time since baseline were included for each participant. While lifespan hippocampal volume is characteristically nonlinear across the lifespan, the linear model was found suitable over the < 5 years considered in this study, and the inclusion of random slopes in addition allowed each participant to have her/his individual rate of atrophy.

### Is there a relationship between memory and hippocampal volume changes following training?

The extent of correlated change in memory performance and hippocampal volume was investigated by fitting two linear regression models separately to each individual; one model for hippocampal volume as a linear function of time since baseline and another model for number of words recalled on the 100-words test as a linear function of time since baseline. The correlation between individual slopes estimated by each of these models was then computed. For these analyses, the final follow-up time point was excluded, in order to only focus on the training period. Next, we computed the correlation between overall memory level and hippocampal volume slope during the training period. The overall memory level was estimated as the intercept of each participant’s memory performance on the 100 words test in an individual regression fit. Finally, we computed the correlation between performance on the 100-words test at follow up and hippocampal volume slope during the training period.

### Is there a transfer effect on a non-trained memory task, and if so, does it relate to the 100-words test performance, and does this persist after cessation of intervention?

A computerized associated word pair task was carried out to test for transfer effects from the intervention. This test has test has previously shown transfer effects in an intervention study using the MoL in both young and older, where transfer effects were related to the improvements on the criterion task^29^. The participants were presented a series of 36-word pairs (two non-associated words) to be memorized as a pair. Each word pair is shown on the screen for five seconds. Next, participants were handed a sheet with a list of words encompassing only one of the words of each word pair. Here, the participants should write down the second word of each word pair. The effect of training on the word pair task was estimated by fitting the model used for the 100-words test (see (“Does the training intervention lead to an increase or maintenance of hippocampal volume relative to that observed with no training, and are such effects observed years after intervention?”section) replacing the 100-words with scores on the word-pair transfer task. The relationship between change on the transfer task and the trained 100-words test was estimated through the correlation of each individual’s linear regression slope. The transfer task was implemented 2 months after the first 23 participants commenced their training. The data were filtered by first including only participant time points containing the 100-words score, and then removing those with less than two (pre and post training) word-pair scores. Thus, a small group (13 young, 10 old) of participants who underwent training but lacked complete assessment on the transfer task was excluded from the analysis.

## Results

### Does the intervention increase memory performance and can effects be observed three years after the cessation of intervention?

**Figure 2:**
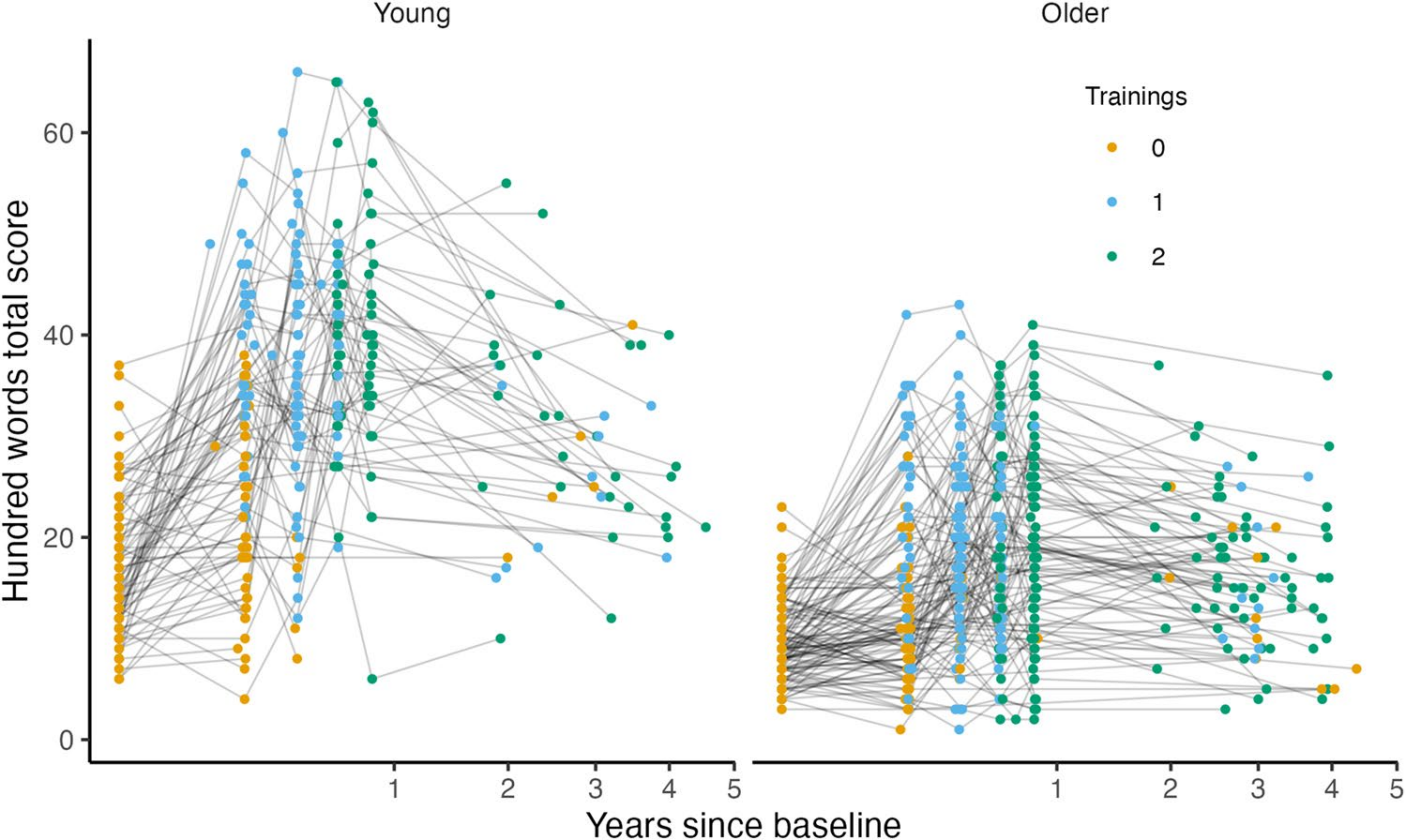
Scores on the 100-words task. The labels show the number of training periods (black = 0, orange = 1, blue = 2) each participant has completed at the given time point, and repeated measurements of a single individual are connected with gray lines. Lines between time-points during the training intervention (within the 1st year) are stretched horizontally, while lines years after cessation (long-term passive period) are compressed for illustrational purposes.

Values on the criterion memory test (100-word test) were compared to baseline after both the first and the second training periods in both age groups. All results are reported with 95% confidence intervals. In the young group, memory performance after the first training period showed an increase of 17.6 (CI 15.7, 19.5) words and 20.6 (CI 18.3, 22.8) words after the second training period. The older group showed an increase of 7.23 (CI 5.76, 8.70) words after the first training period, and 8.11 (CI 6.42, 9.80) words after the second training period. See Tables 4 and 5 (on page 28) for scores at each time point. The retest effect was estimated to an increase of 2.80 words (CI 1.50, 4.09). There was a notable effect of age within the older age group, estimated to a decrease of − 0.54 words (CI − 0.90, − 0.17) per additional year. For the young group, the age estimate was − 0.08 words yearly (CI − 0.52, 0.36). See Fig. 2. For further details, see Supplementary Table S1.

**Table 4:** Scores on the hundred words task (criterion task) and the word pair task (transfer task) of young and older training groups at all time points, separated by group allocation according to the ABAB/BABA design.

		Tp0 Pre rest 1 (only for BABA)		Tp1 Pre intervention 1		Tp2 Post intervention 1		Tp3 Pre intervention 2		Tp4 Post intervention 2		TP5 Post rest 2 (only for ABAB)		TP6 follow-up	
		Mean	SD	Mean	SD	Mean	SD	Mean	SD	Mean	SD			Mean	SD
BABA		15F/18 M		14F/16 M		10F/9 M		8F/7 M		7F/5 M				9F/7 M	
Start rest young	Hundred Words	18.3	7.48	20.4	9.71	38.2	13.3	40.4	11.0	44.6	10.7			32.7	12.0
	Word pairs	18.1	10.1	20.0	9.46	22.8	8.17	26.1	5.69	30	4.31			26.8	7.34
		37F/17 M		35F/16 M		31F/11 M		31F/11 M		31F/10 M				27F/11 M	
Start rest old	Hundred Words	9.20	4.08	10.6	5.42	17.6	7.80	18.2	8.42	17.5	9.28			15.2	6.77
	Word pairs	5.13	4.61	7.39	5.99	5.46	5.27	8.58	5.25	10.5	8.78			7.06	5.95

ABAB	Sex			27F/20 M		19F/15 M		18F/14 M		18F/10 M		16F/10 M		15F/12 M	
Start train Young	Hundred Words			16.3	6.75	38.1	9.29	34.9	10.8	39.8	10.1	37.3	12.1	27.7	9.14
	Word pairs			19.1	8.89	22.2	7.96	23.6	8.03	20.7	6.42	24.5	7.85	23.0	8.78

				28F/31 M		25F/21 M		21F/21 M		21F/20 M		20F/20 M		24F/8 M	
Start train old	Hundred Words			9.47	3.28	20.0	9.91	20.2	8.88	20.8	10.2	22.8	9.39	18.3	8.06
	Word pairs			3.96	3.96	7.95	6.57	11.1	8.38	10.3	8.49	11.2	8.37	9.34	6.92

**Table 5:** Scores on the hundred words task (criterion task) and the word pair task (transfer task) of young and older active control groups at all time points, separated by group allocation according to the ABAB/BABA design.

Active controls		Tp1 Pre active control		Tp2 Post active control		Tp3 Pre train 1		Tp4 Post train 1		TP6 follow-up	
		Mean	SD	Mean	SD	Mean	SD	Mean	SD	Mean	SD
		17F/2 M		11F/2 M		3F/2 M		2F/2 M		3F/2 M	
Active control young	Hundred Words	16.1	5.15	24.5	6.33	18	9.72	37.5	8.58	27.4	3.44
	Word pairs	18.9	8.81	19.2	8.60	21.8	4.60	21.8	3.40	25	4.55

		13F/7 M		12F/7 M		8F/4 M		8F/4 M		9F/4 M	
Active control old	Hundred Words	9.35	3.96	10.1	3.84	11.1	3.29	18.2	7.11	14.2	5.49
	Word pairs	7.3	5.55	9.21	5.78	6.75	4.97	6.25	7.10	6.38	5.45

**Figure 3:**
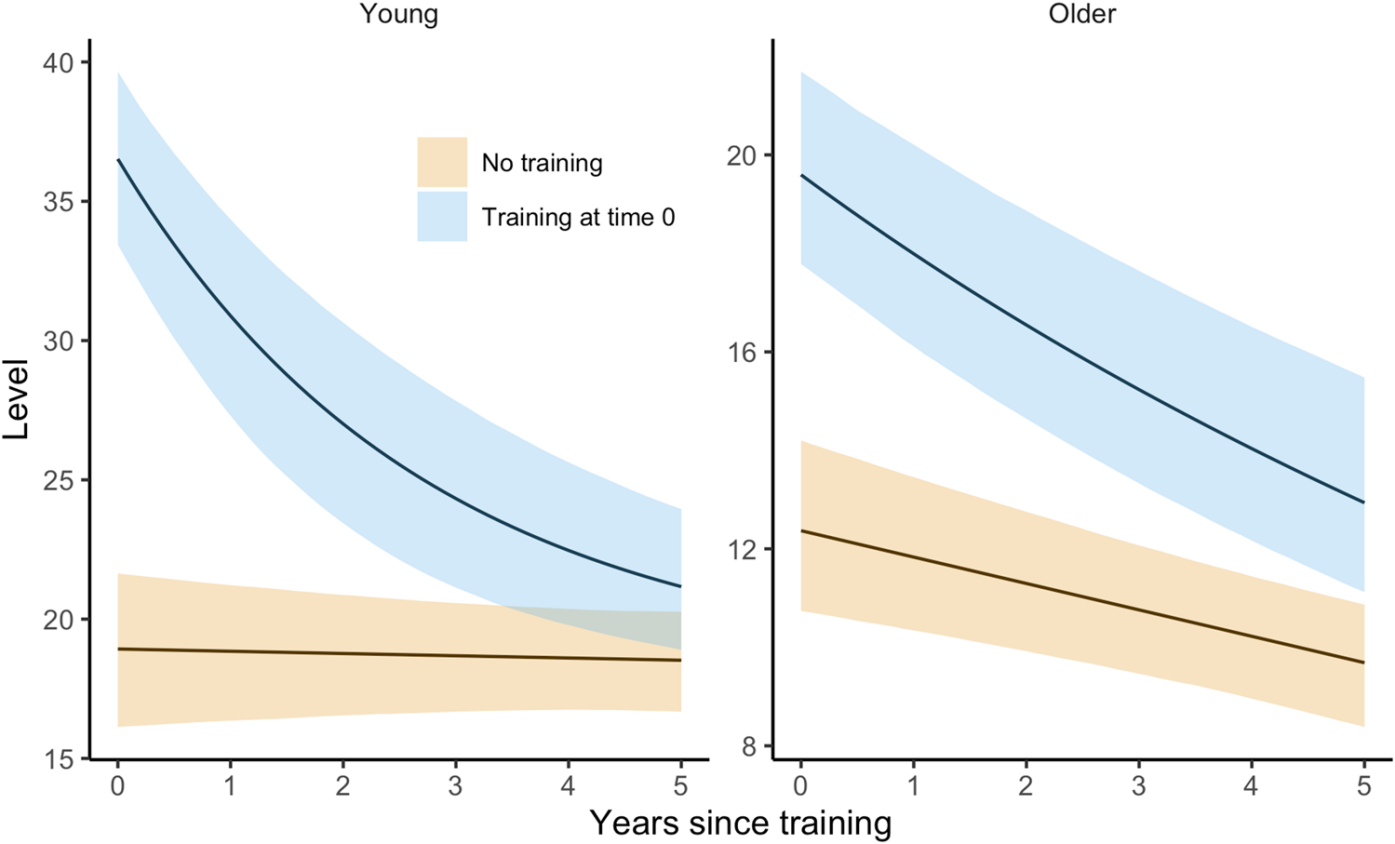
Estimated change in memory performance in the years following the training.

To estimate the degree of maintenance of training effect on memory over several years, an exponential term was computed including the estimated values of the first training effect and decay across time intervals passed since last training period. The effect of training was estimated to prevail for at least 4.5 years in the young and 6.5 years in the older group, see Fig. 3. However, effects beyond the follow-up period are uncertain. For estimations of long-term effects after even longer time intervals, see Supplementary Fig. S1.

### Does the training intervention lead to an increase or maintenance of hippocampal volume relative to that observed with no training, and are such effects observed years after intervention?

**Figure 4:**
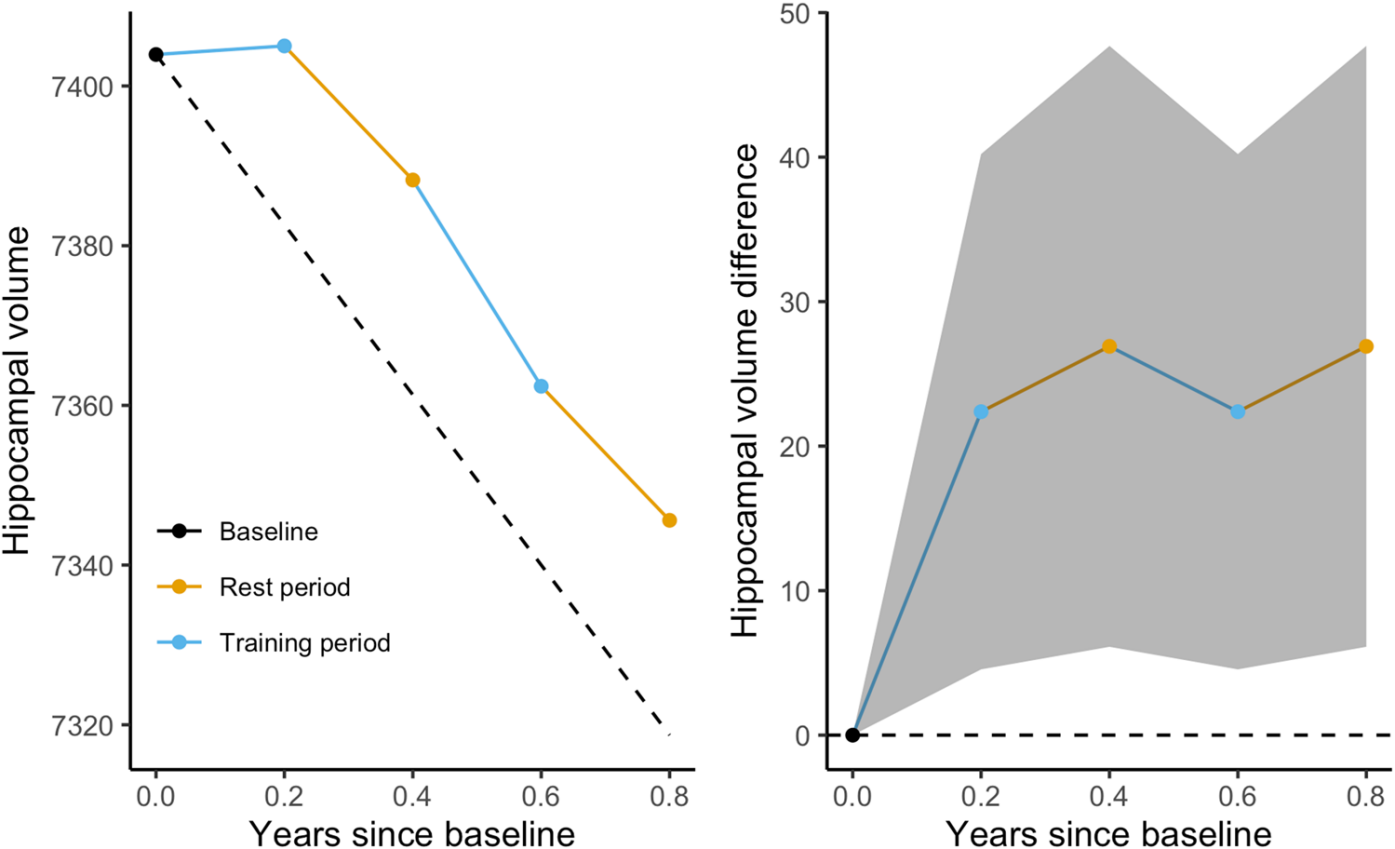
Estimated short-term effect of the training in the older group. The dashed line represents the reference condition of no training. The right parts of the plots show the estimated difference between the curves, with 95% confidence intervals for the difference.

In the young adults, the linear mixed model showed a non-significant relative increase in hippocampal volume of 10.3 (CI − 14.0, 34.6) mm^3^ directly after training, and a significant total relative increase of 28.5 (CI 1.2, 55.8) mm^3^ after ten passive weeks. The older group showed significant total relative increase of 22.4 (CI 4.6, 40.2) mm^3^ directly after training and 26.9 (CI 6.2, 47.6) mm^3^ after the ten passive weeks, see Fig. 4. For further details of the effects on hippocampal volume, and prevailing effects, Supplementary Table S3. For individual slopes of hippocampal volume throughout the intervention, see Supplementary Fig. S5. When compared to a passive control group after a long-term passive period of 3 years, we found no evidence of difference in hippocampal volume between those who had trained and those who did not train, with estimated differences − 77 (CI − 314, 160) mm^3^ in the young trainers relative to non-trainers and a non-significant difference of 136 (− 83, 355) mm^3^ in favor of older trainers relative to older non-trainers.

### Is there a relationship between memory and hippocampal volume changes following training?

No correlations were observed between change in hippocampal volume and change in memory performance during training, between slope of hippocampal volume during training and overall memory performance, or between hippocampal volume slope during training and performance on the 100-words test at follow-up in any of the age groups.

### Is there a transfer effect on a non-trained memory task, and if so, does it relate to the 100-words test performance, and does it persist after cessation of intervention?

**Figure 5:**
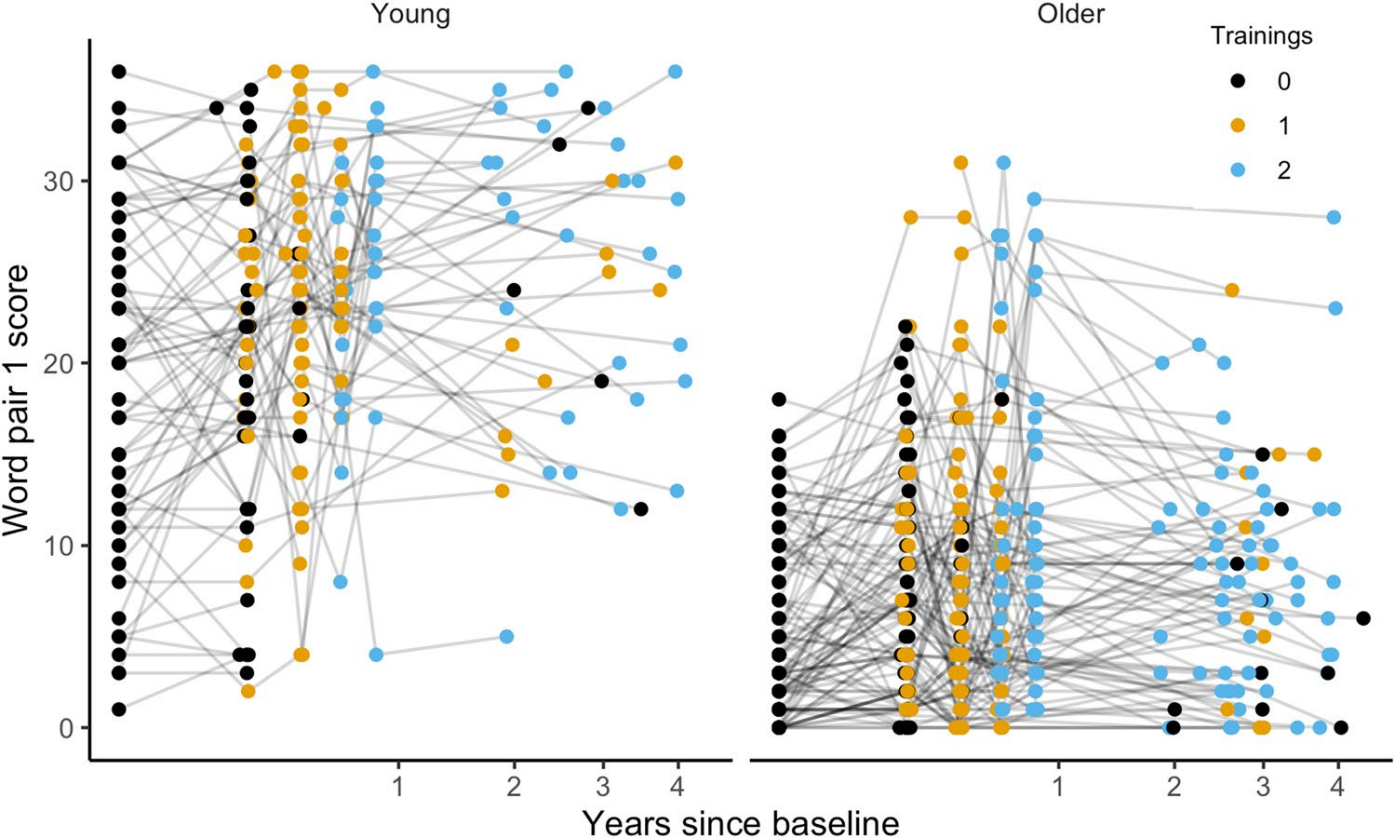
Scores on the word-pair task. The labels show the number of trainings (none, 1, or 2) each participant has completed at the given time point. Repeated measurements of a single individual are connected with gray lines. Lines between training time-points (within the first year) are stretched horizontally, while lines years after cessation (long-term passive period) are compressed for illustrational purposes.

Values on the transfer task (word-pair association task) were compared to baseline after both the first and the second training period in both age groups. In the young group, performance after the first training period showed significant increases of 1.89 (CI 0.35, 3.42) correct word pair associations and 3.94 (CI 1.79, 6.09) correct word-pair associations after the second training period. The older group showed a non-significant increase of 0.37 (CI − 0.86, 1.60) after the first training period and a significant increase of 2.36 (CI 0.78, 3.95) word-pairs after the second training period, see Fig. 5. For further details on the estimation of the effects on the transfer task, see SI, Supplementary Table S2.

**Figure 6:**
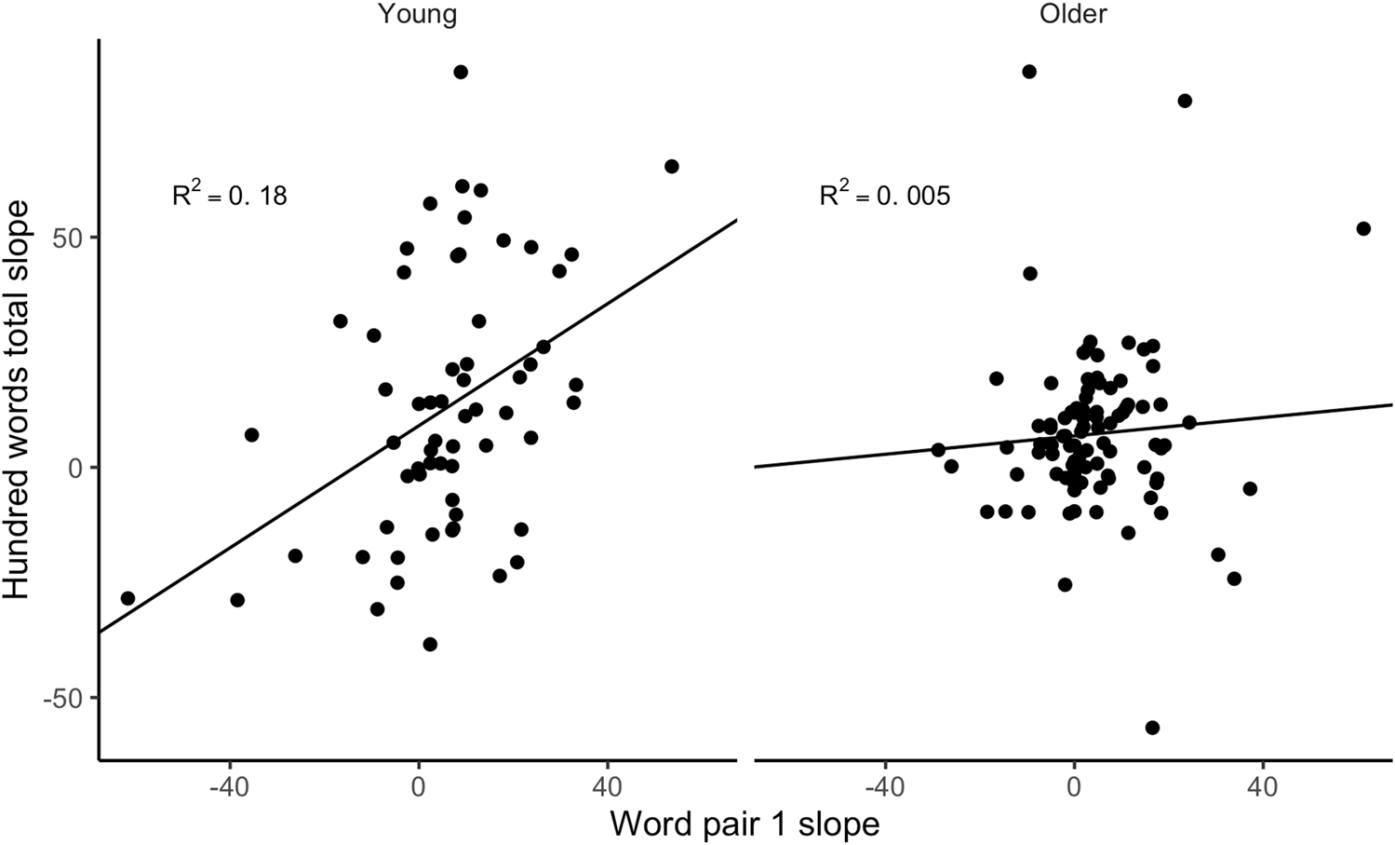
Correlation between the slopes on the 100-words test and the word-pair test.

During the training period, there was a positive correlation of 0.42 (CI: 0.19, 0.61) between individual slopes of number of words recalled on the 100-words test and correct word-pair associations in the young group. No such relationship was seen in the older group, where the estimated correlation was 0.07 (CI − 0.13, 0.27). See Fig. 6. For values on the criterion task and the transfer task at each time point for the groups separately, see Tables 4 and 5.

## Discussion

We investigated the immediate and long-term effects of memory training on memory performance and hippocampal volume in young and older healthy adults. The training yielded durable increases in memory performance on a criterion task, which persisted for several years. During the intervention, older adults showed a relative increase in hippocampal volume, which was maintained 10 weeks after the conclusion of the intervention. Similar increases in hippocampal volume were observed 10 weeks after the intervention in the young group. However, when compared to a passive control group at 3 years follow-up, total hippocampal volume of the memory trainers did not differ from that of the controls in any of the age groups. Change in hippocampal volume during the training period did not relate to memory increases during training, to overall memory performance during the whole study, or to memory performance at the follow-up time point.

In the young, but not older group, a transfer effect was observed on a non-targeted memory task, which was positively related to the improvement on the trained memory task. Both young and older participants showed a significant increase in memory performance immediately after the training intervention, after ten passive weeks, and 3 years after the intervention. This is in line with maintained training effects on memory performance in older adults from interventions applying usage of the MoL^15,47^. In these studies, the training effects were observed in older adults both 6 months and 3, 5 years after the intervention^47^. Long-lasting effects have additionally been observed 2 years after completing a multi-domain training intervention^48^.

The older adults showed an increase in hippocampal volume during the memory-training periods, which prevailed 10 weeks after conclusion of the training. Increases were also observed in the young 10 weeks after conclusion of the training. Contrary to the upheld memory gains, the effects on hippocampal volume had subsided after 3 years. The first study to report plastic brain-changes after a training intervention showed that practicing juggling induced gray matter changes in young volunteers that retracted after a 3 months passive period^49^, suggesting a dependency of continuous training. This aligns with previous results from the present study where we found that training-specific changes in white matter was dependent on continuous training during the periods of on-and-off training^50^. In terms of training benefits on the hippocampus, one study has shown training related gains in navigation performance and stable hippocampal volumes that were maintained 4 months after termination of training (Lövdén, Schaefer et al. 2012). The authors suggested that sustained cognitive demands protect hippocampal integrity against age-related decline. Immediate effects on the hippocampus in the navigation study were observed in both young and older. Here, immediate hippocampal volume effects were only found in the older, while both age groups showed significant prolonged increases 10 weeks after the training. The reason for this discrepancy is uncertain, but this could relate to a difference in power between the age groups in the present study (57 young, 100 older). However, the absolute effects appear larger in the older group. It should be noted that the older participants in our study were on average about a decade older than those studied in the navigation intervention^51^. Compared to the navigation study, difference of intervention, the presently longer follow-up interval, or both may also have played a role. In addition, it may well be that the present memory intervention was subjectively more challenging to the older adults, or that its effects got more pronounced in the context of accelerated volumetric decline with age. Our training-dependent effect on hippocampal volume indicates that the training could to a certain degree have counteracted age-related hippocampal decline during training, and that this can be upheld for several months. However, we found no difference between the training group and the passive control group after 3 years of no training, suggesting that the cognitive demands need to be upheld to maintain the training benefits on hippocampal volume over several years. Given that no assessments were carried out between the short-term follow up after ten passive weeks and the long-term assessments after 3 years, the potential for maintenance beyond 10 weeks is unknown.

Although we have previously found a relationship between white matter change and memory benefits from the training intervention^18,50^, no relationship was found between change in memory performance and hippocampal volume change during training. While it seems reasonable that brain changes during training should relate to individual differences in cognitive changes, the present null-finding is in accordance with previous literature on change-change relationships, as such have not invariably been observed^52^. It is, however, uncertain whether a longer intervention would have shown different results. Partially in line with the plasticity-flexibility framework^30^, upheld volumetric benefits in the hippocampus did not appear to be a requirement for maintenance of cognitive improvements. This suggests that, at least for the majority, the resources required for long-term maintenance of training benefits to cognitive performance did not exceed that of the participants´ already available range of flexibility.

Outcome from memory-training commonly shows large individual differences at all ages, and these are often magnified with increasing age^50,53,54^. Here, immediate training effects on the brain were only present among the older participants, perhaps suggesting a greater reliance on brain structure change in the older group than in the young during training. We have previously reported that individual variation in task completion did not affect the training effects in our study^18^ However, this is a limitation to the study that should be taken into account in the development of future training programs. While the group-size discrepancies may affect the results, it could also be that the young participants found the training less challenging, and they may have had lower motivation as their memory was better to begin with. Level of novelty and suspense has been observed to affect motivation in educational video games^55^, which suggests that these factors could be of importance to maintain the interest in the young participants, possibly both in terms of type of task or memory process measured, and in structure of the intervention, between-level goals or environment, or similar. As the older participants show great variation in trajectory and amount of improvement, such factors may also be beneficial for those that perform the best amongst also the elderly, offering an additional possibility for individual adaptations beyond mere age-related adjustments of difficulty.

Great attention, albeit with discrepant conclusions, has been given to whether the outcome of cognitive training is task-specific, or transferable to other cognitive abilities^25,26,56–59^. We observed improvement in the young group on a non-trained word-association task implemented to test for transfer effects. Those who benefited the most from the memory-training on the criterion task were also the ones with the greatest improvement on the transfer task. While the transfer task differed from the criterion task in terms of memory process targeted, it includes components of episodic memory and requires visualization abilities, which are likely improved using the MoL. Thus, while we do not suggest far-transfer effects, we note that some changes in performance were seen beyond the increase in rendition on the strictly training-specific task.

The sample showed a mean IQ above average, which is not unusual in samples of voluntary participation^60^. IQ has repeatedly been related to learning abilities in adults^61–63^, and we have previously reported that IQ was positively related to memory-training outcome in the present study^64^. Here, IQ was also associated with study attrition, as is commonly seen it these types of studies^37^. A total of 23 participants dropped out, and the drop-out analysis showed a bias towards a higher cognitive functioning in those who did not drop out. The degree and mechanisms through which IQ may have affected the training outcome remain unknown, and the selection bias is acknowledged as a limitation of the study. While this is challenging to avoid, there are many potential causes of drop-outs beyond the attrition bias which can be taken into account in future studies. The participants partook in weekly face-to-face group meetings, which requires a great amount of travel time and schedule restrictions for the participants, as well as resources from the intervention team. Indeed, the participants who dropped out reported that the fixed meetings schedules were inconvenient^35^. It is possible that a more flexible training intervention could be beneficial to avoid attrition effects. A recent study by Sandberg et al.^65^ developed a mobile phone application, enabling the participants to carry out MoL tasks on their phone. In correspondence with our results, the participants showed great memory benefits across ages. Thus, an interesting approach for future research is to make greater use of the currently available technology. This could facilitate adjustments to ensure a proper challenge and motivation level, increasing compliance and ensuring continuous training. However, a social component to training may in itself also increase motivation and function^66–69^ .

Our results convey that memory training can lead to maintained benefits in memory performance after 3 years, and that effects on hippocampal volume can be maintained at for some months, but not years, following cessation of training. Thus, while training produces long-term effects on memory performance, structural benefits on hippocampal volume over years may be reliant upon continuous training. It is of special interest for future research to further investigate the possible premises for efficient memory-training and effects on both memory and hippocampal volume, in order to adjust individual training interventions accordingly. This could effectuate a positive impact on general memory performance and hippocampal measures at all ages, in addition to counteracting age-related neuropsychological decline.

## Supplementary Information

Supplementary Information.

## Data Availability

The data are available from the corresponding author upon reasonable request, given appropriate ethical and data protection approvals. R code for running all the analyses is available at the GitHub repository https://github.com/LCBC-UiO/memory-training-ncp, where we also provide simulated datasets which can be used to run the code.
