# Supplementary material for: Cognitive and hippocampal changes weeks and years after memory training
Source: Sci Rep. 2022 May 12;12:7877. doi: 10.1038/s41598-022-11636-4 (PMC9098907; doi:10.1038/s41598-022-11636-4)
Supplement: Supplementary file 1 — Supplementary Information. [file 41598_2022_11636_MOESM1_ESM.pdf]

Supplementary Information

Title

Cognitive and hippocampal changes weeks and years after memory training

Authors

Anne Cecilie Sjøli Bråthen*[1], Øystein Sørensen[1], Ann-Marie Glasø de Lange[1,3], Athanasia M. Mowinckel[1], Anders M Fjell[1,2], Kristine B Walhovd[1,2]

The data are available from the corresponding author on reasonable request, given appropriate ethical and data protection approvals. R code for running all the analyses is available at the GitHub repository https://github.com/LCBC-UiO/memory-training-ncp, where we also provide simulated datasets which can be used to run the code.

1. Supplementary Methods

Follow- up intervals

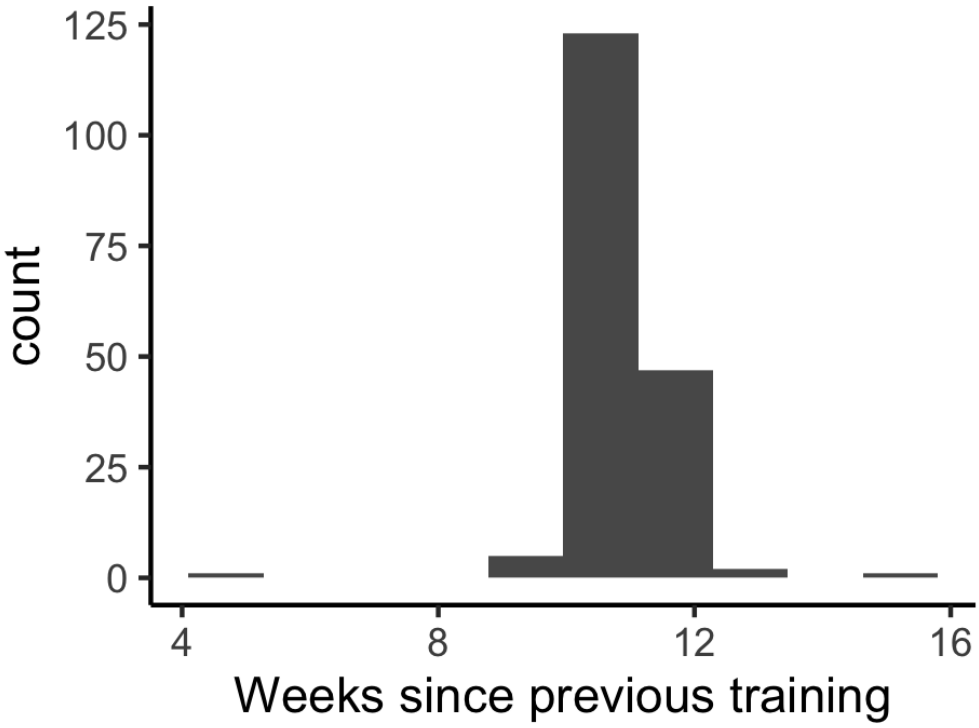

Supplementary Fig. S1. Distribution of time since training at short-term follow up (after 10-12 weeks). The histogram shows the prevailing effect of training after 10-12 weeks.

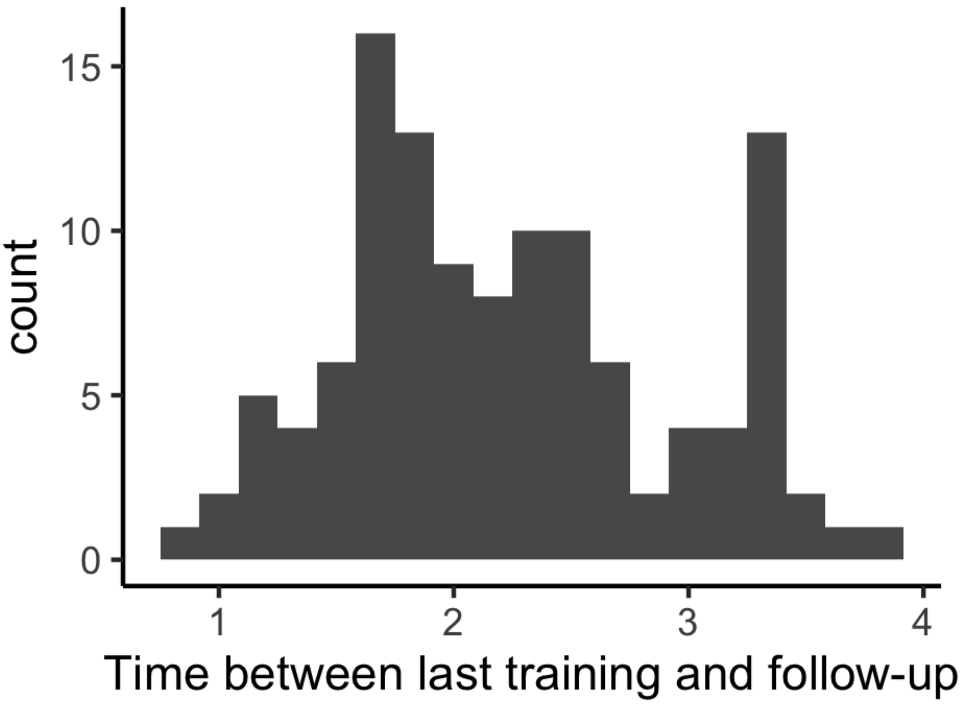

Supplementary Fig. S2. Time between last training and follow-up for all participants.

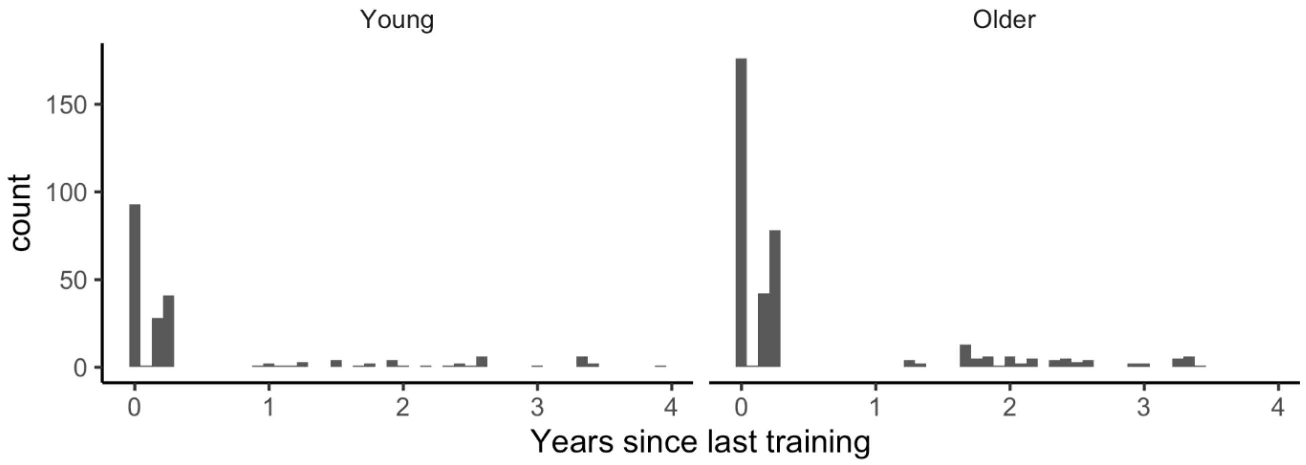

Supplementary Fig. S3. Time since training for each observation. Time points prior to which no training has been completed have been removed. The peak at zero corresponds to the time points immediately after a training session, and the long tail to the right corresponds to the follow-up time points.

2. Supplementary results

2.1 Immediate effects on memory training task

**Age group**	**Variable**	**Estimate**	**95% CI**	**p-value**
**Young participants**	Intercept	18.5	16.7, 20.4	0.176
	Age slope	-0.08	-0.52, 0.36	0.718
	1st training	17.6	15.7, 19.5	2.2e-61
	2nd training	20.6	18.3, 22.8	1.43e-59
	Exp. decay	4.41	2.87, 5.96	3.7e-08
**Older participants**	Intercept	9.69	8.12, 11.3	4.8e-33
	Age slope	-0.54	-0.90, -0.17	0.005
	1st training	7.23	5.76, 8.70	1.4e-20
	2nd training	8.11	6.42, 9.80	8.7e-20
	Exp. decay	1.86	0.17, 3.55	0.032
**Common effect**	Male - female offset	-1.85	-3.64, -0.07	0.043
**Common effect**	>=1 retest	2. 80	1.50, 4.09	2.8e-05

Supplementary Table S1. Parameters of correct words on 100-words task after first and second training.

Estimations of long-term effects on memory training task at a follow-up

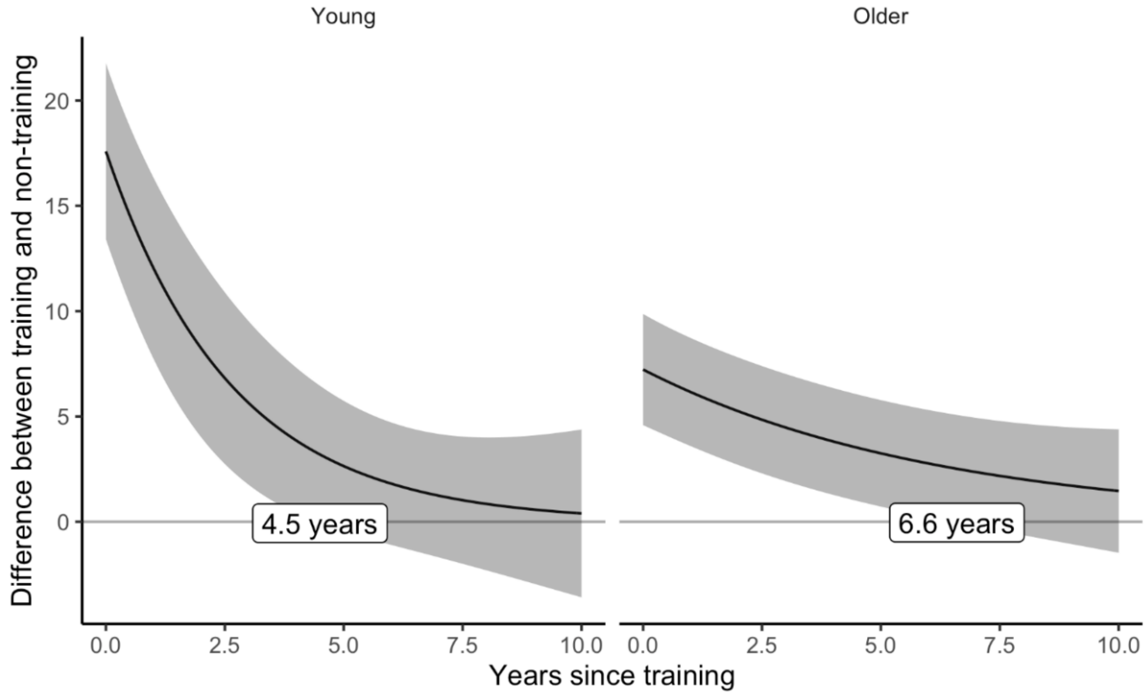

Supplementary Fig. S4. The effect of training was estimated to prevail at least for 4.5 years in the young and 6.6 years in the older (see Manuscript; Results 4.1). For reference, we provide a plot of the estimated difference between the training and non-training group up to ten years after

training. In the older participants, the 95 % confidence interval goes below zero after 6.6 years. We do however re-emphasize that we should be cautious in interpreting these estimates based on extrapolation beyond 4 years.

2.2 Transfer effect on a non-trained memory task

Age group	Variable	Estimate	95% CI	p-value
**Young participants**	Intercept	19.5	17.8, 21.2	1.6e-13
	Age slope	-0.13	-0.54, 0.29	0.547
	1st training	1.89	0.35, 3.42	0.017
	2nd training	3.94	1.79, 6.09	3.8e-04
	Exp. decay	-0.30	-2.87, 2.27	0.818
**Older participants**	Intercept	6.22	4.78, 7.66	4.3e-18
	Age slope	-0.58	-0.92, -0.25	8.0e-04
	1st training	0.37	-0.86, 1.60	0.560
	2nd training	2.36	0.78, 3.95	0.004
	Exp. decay	1.66	-3.24, 6.56	0.510
**Common effect**	Male - female offset	-1.94	-3.63, -0.26	0.025
**Common effect**	>=1 retest	1.88	0.84, 2.93	4.9e-04

Supplementary Table S2. Estimated parameters of transfer task after first and second training

2.3 Effect of memory training on hippocampal volume

Description	Estimate	95% CI	p-value
Intercept young participants	8476	8347, 8605	3.1e-247
Intercept older participants	7404	7281, 7527	5.5e-237
ICV (1 standard deviation)	321	232, 410	1.5e-11
Male-female diff	-59.3	-243.2, 125	0.528
Slope young participants	-32.6	-43.3, -21.9	3.7e-09
Slope older participants	-106.6	-116.8, -96.3	2.4e-74
Offset Young participants directly after training	10.3	-14.0, 34.6	0.408
Offset Older participants directly after training	22.4	4.6, 40.2	0.014
Offset Young participants after 10 passive weeks	28.5	1.2, 55.8	0.042
Offset Older participants after 10 passive weeks	26.9	6.2, 47.6	0.011

Supplementary Table S3. Parameter estimations of hippocampal volume change (mm3).

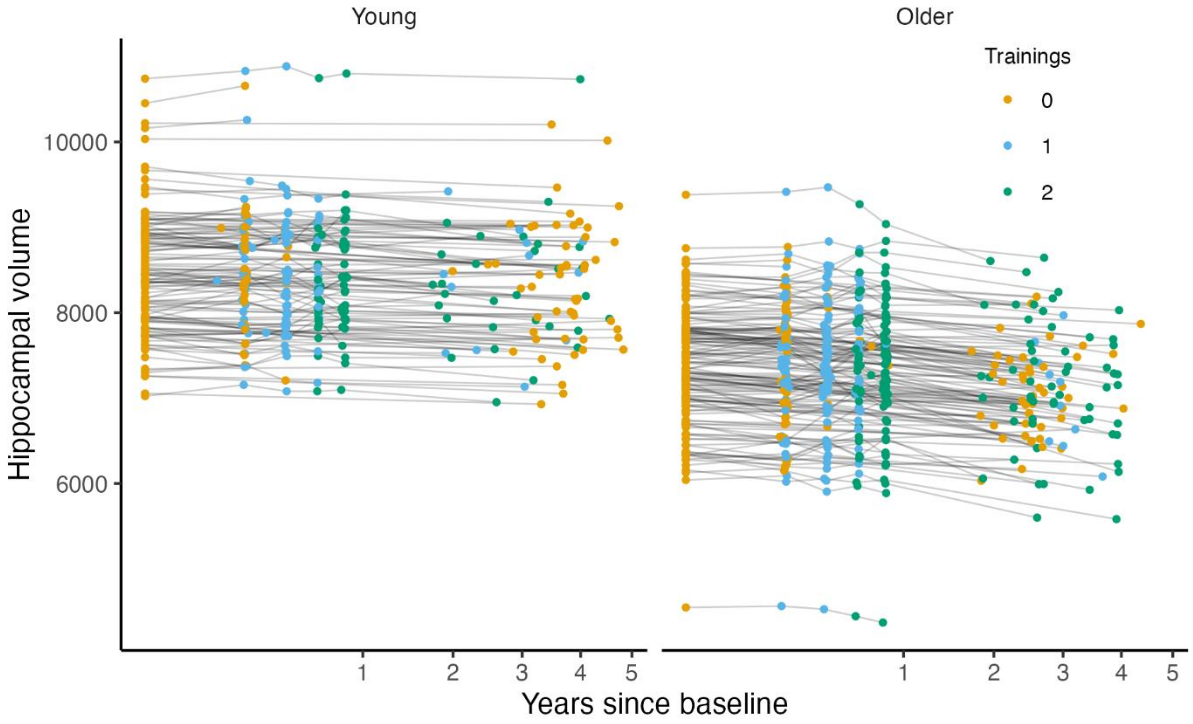

Supplementary Fig S5. Individual slopes on hippocampal volume throughout the study.
